# Fecal Lactoferrin: Reliable Biomarker for Intestinal Inflammation in Pediatric IBD

**DOI:** 10.1155/2015/578527

**Published:** 2015-05-24

**Authors:** Stephan Buderus, James H. Boone, Michael J. Lentze

**Affiliations:** ^1^Department of Pediatrics, St. Marien-Hospital, GFO-Kliniken Bonn, Robert-Koch-Straße 1, 53115 Bonn, Germany; ^2^TechLab Inc., Blacksburg, VA 24060, USA; ^3^Department of Pediatrics, University Children's Hospital, Adenauerallee 119, 53113 Bonn, Germany

## Abstract

*Background*. Optimal management of pediatric patients with inflammatory bowel disease (IBD) requires early diagnosis. Aim of the study is to compare fecal lactoferrin (FL) as biomarker of intestinal inflammation to CRP in pediatric patients with new-onset IBD.* Methods*. FL was measured by ELISA in stool specimens collected prior to endoscopy for IBD (IBD-SCAN; TechLab, Blacksburg; normal < 7.3 *µ*g/g feces). CRP was detected in serum (normal < 5 mg/L). Three patient groups were determined: *n* = 21 (mean age 13.2) with Crohn's disease (CD), *n* = 15 (mean age 10.9) with ulcerative colitis (UC), and *n* = 20 (mean age 11.9) in whom IBD was ruled out. In CD patients the endoscopic severity score SES-CD was correlated with the FL levels.* Results*.* (Mean ± SEM)*. CRP levels were 27.18 ± 4.2 for CD-cases, 20.8 ± 9.5 for UC, and 0.24 ± 0.06 for non-IBD patients. FL levels were 313.6 ± 46.4 in CD, 370.7 ± 46.9 in UC, and 1.3 ± 0.5 in non-IBD patients. Sensitivity of CRP to detect IBD was 75% with specificity of 100%, positive predictive value of 100%, and negative predictive value of 69%. Sensitivity of FL was 100% with specificity of 95%, positive predictive value of 97.3%, and negative predictive value of 100%. In CD, FL levels correlated positively (*R* ^2^ = 0.42) with disease severity as judged by the SES-CD.* Conclusions*. Elevated FL corresponds to intestinal inflammation, even in patients with normal CRP. With high probability, normal FL excludes intestinal inflammation.

## 1. Background

Inflammatory bowel disease (IBD) involves a large clinical spectrum of disease presentations from mild to severe symptoms in relation to different disease locations and the extent from possible rectal to upper intestinal involvement. In pediatric patients, early diagnosis of IBD is of great relevance and essential for best outcome. Induction of remission by specific therapy aims to improve the patient's symptoms, to maintain or restore the quality of life as soon as possible, and to prevent complications of the disease [1–5]. Human lactoferrin, a neutrophil derived glycoprotein, can be measured in feces and whole gut lavage as an indicator of intestinal inflammation in both IBD and infectious gastroenteritis [6, 7]. Recent studies have shown fecal lactoferrin (FL) as a sensitive biomarker for pediatric IBD [8, 9]. In addition, this biomarker can serve as an aid for guiding the diagnostic and therapeutic process for both pediatric and adult IBD [10–12]. In this study, we evaluated the utility of FL compared to CRP [13, 14] for diagnosing pediatric IBD characterized by endoscopic and histologic examination. Moreover, we analysed FL in comparison to the disease severity in CD as defined by the endoscopic score SES-CD [15].

## 2. Patients and Methods

### 2.1. Patients

In this observational study 56 pediatric patients who qualified for colonoscopy because of symptoms suggestive of IBD were recruited. Ileocolonoscopy with biopsies was performed by an academic teaching hospital, serving as tertiary care pediatric gastroenterology center. As part of the routine clinical assessment, CRP and the FL were determined in all patients prior to endoscopy. Based on results of endoscopy and histology, the diagnosis of IBD was either established or ruled out as non-IBD. The IBD patients were further defined as Crohn's disease (CD) or ulcerative colitis (UC). The study was performed with the approval of the local ethical committee.

### 2.2. Data Collection and Laboratory Analysis

Fecal lactoferrin was determined quantitatively by an ELISA (IBD-SCAN; TechLab, Blacksburg, VA) in stool specimens collected prior to endoscopy as part of the diagnostic workup for IBD. FL results are reported as *μ*g/g feces (normal < 7.3). Serum-CRP was determined by standard methods and is reported as mg/L serum (normal < 5). Inflammatory bowel disease was diagnosed and classified according to the “Porto-Criteria,” that is, based on the clinical picture, laboratory, and imaging results and most importantly on the results of endoscopy and histology [16]. All patients had an ileocolonoscopy with biopsies taken from each examined segment of the intestine. The macroscopic aspect of the ileocolon was described qualitatively as normal, or showing slight, intermediate, or severe inflammation, respectively; in addition the SES-CD [15] was calculated as endoscopic severity score in CD patients. The score evaluates size of ulcers, ulcerated surface, affected surface, and narrowing in the different segments of the colon and terminal ileum, with a maximal score of 60. In addition, the distribution of the disease was categorized according to the “Paris Classification,” the pediatric modification of the Montreal Classification [17]. According to the Porto-Criteria, all patients with new-onset IBD also received an upper endoscopy and small bowel imaging.

### 2.3. Statistical Analysis

Calculations and plotting of the graphs were performed using GraphPad Prism, version 4.03 for Windows, GraphPad Software, San Diego, California, USA. Results are expressed as mean ± standard deviation (SD), unless otherwise indicated. In the box and whiskers graphs, the mean is depicted by the line in the box which extends from the 25th to the 75th percentile of the data. Significance levels to compare CRP and FL of the IBD patients versus controls were calculated using the two tailed *t*-test, with a *P* value <0.05 set as significant.

## 3. Results

**Figure 1:**
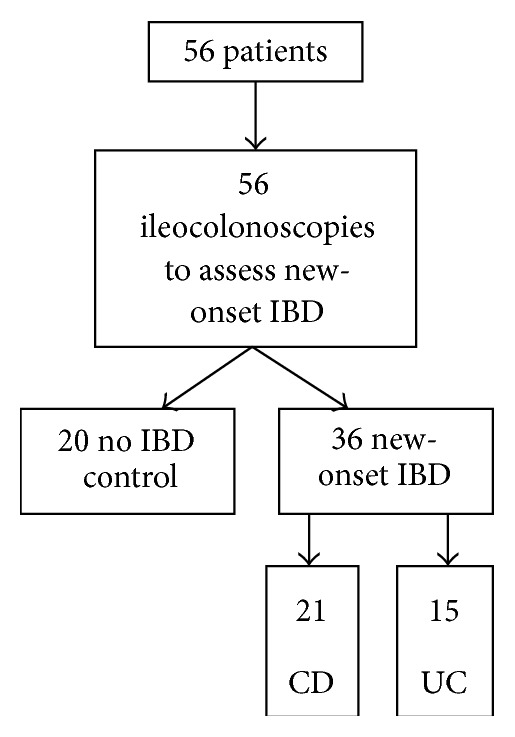
Patient and endoscopy flowchart.

A total of 56 patients with ileocolonoscopies were included in this study (Figure 1). The demographic and clinical characteristics are shown in Table 1. There were 20 patients with non-IBD illnesses including functional abdominal pain, irritable bowel syndrome, or constipation. For these patients, upper and lower endoscopy and the histology of both examinations were normal with no signs typical for IBD.

### 3.1. CRP

**Figure 2:**
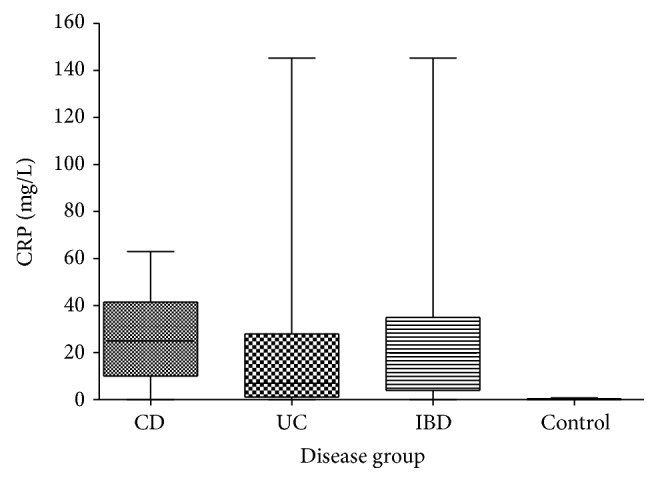
Serum-CRP levels stratified for groups of patients. *P* < 0.0001 IBD versus control and CD versus control. *P* = 0.017 UC versus control. *P* = 0.50 for CD versus UC.

In the IBD group, the mean CRP levels were elevated with a wide range of concentrations (Figure 2). The mean CRP for CD cases was 27.2 mg/L (range 0–63.00; SD 19.04); for UC cases, the mean CRP was 20.8 (range 0–145.2; SD 36.63). There were 2 CD (9.5% of CD) patients with normal CRP. Accordingly, 3 out of 15 UC (20%) patients had negative CRP. All of the UC patients with CRPs ≥ 16 mg/L suffered from severe pancolitis. None of the non-IBD patients had elevated CRP with a mean of 0.24 mg/L (SD 0.28) and a maximum of 0.8 (Figure 2). Sensitivity of CRP to detect IBD was 75% with a specificity of 100%, correspondingly resulting in a positive predictive value of 100% and negative predictive value of 69%; the negative likelihood ratio (LR) of CRP is 0.25.

### 3.2. Fecal Lactoferrin (FL)

**Figure 3:**
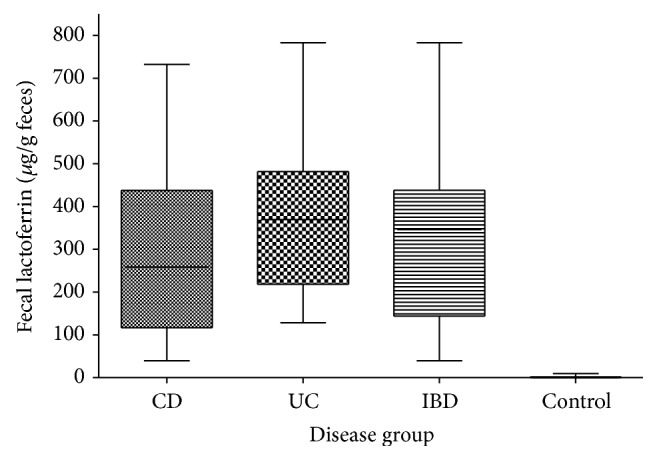
Fecal lactoferrin levels stratified for groups of patients. *P* < 0.0001 for each IBD, CD, and UC versus control. *P* = 0.41 for CD versus UC.

**Figure 4:**
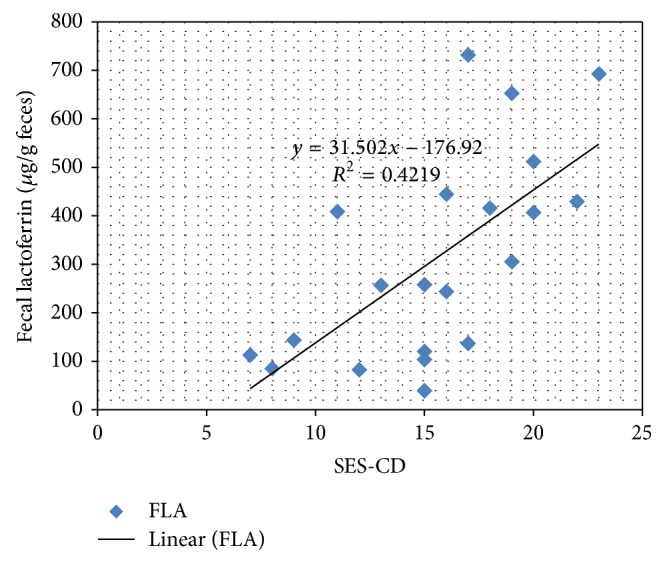
CD patients: fecal lactoferrin and the endoscopic score SES-CD, linear regression line depicted in the graph.

The mean levels of FL for the IBD patients were 314 *μ*g/g CD (SD 212.8) and 371 *μ*g/g UC (SD 181.5) whilst mean FL in controls was only 1.3 (SD 2.4) (Figure 3). Only a single non-IBD-patient had an elevated FL of 9.6 *μ*g/g feces, slightly above the clinical cut-off of 7.3 ug/g. Therefore, sensitivity of FL was 100% with a specificity of 95%, the positive predictive value being 97.3% and having a negative predictive value of 100%. The positive LR for FL is 20.0, whilst the negative LR is 0. For the CD patients the results of the endoscopic severity index SES-CD in correlation with the corresponding FL levels are depicted in Figure 4. There is a positive correlation of CD-SES and FL, showing higher levels of FL (*R* ^2^ = 0.42 and *P* = 0.014) in those patients with a more severe disease as judged by endoscopy.

## 4. Discussion

These results demonstrate the value of determining fecal lactoferrin levels as an aid for diagnosing IBD in pediatric patients and correlate levels with disease severity as judged by the SES-CD in CD patients. Elevated FL (≥7.3 ug/g feces) differentiated precisely between subjects with the presence of intestinal inflammation and those having intact and normal intestinal; this is underscored by the high positive (20) and low (0) negative LR of FL. Patients with normal FL had no evidence of microscopic or histologic intestinal inflammation. This is in accordance with other pediatric and adult studies [7–9, 11]. The presence of elevated LF in patients with normal CRP levels during active disease shows the inferiority of this parameter in comparison to FL to detect IBD. Currently, serum-CRP is an inflammatory serological marker that is routinely measured in a number of clinical situations, among them IBD. The strength of CRP is that every physician can easily and rapidly obtain a result as a routine test. Previous studies have shown [13, 14, 18] that, in CD, CRP is useful in identifying new patients with active disease and monitoring a response to therapy. A limitation for CRP measurements is that levels may be normal in up to 24–64% of pediatric IBD patients experiencing a flare, making it insufficient for excluding active IBD. This corresponds well to our findings that 10% of our active CD patients and 20% of the UC patients were CRP-negative.

A new finding from this study is the observation that the levels of LF correlate with disease severity. In CD patients grouped by SES-CD those with more severe disease had significantly higher LF levels than patients with less inflammation. In UC, we could not observe significant differences between LF levels as all patients had extensive colitis (Paris E4 and Paris E3).

A weakness of the study with respect to parameters such as sensitivity or specificity is the fact the study group is preselected for suspicion of IBD in a tertiary center. This means that those parameters might be lower in a less restricted patient group. But we have clearly shown in a relatively large control group with symptoms severe enough to justify endoscopy that normal FL excludes intestinal inflammation caused by IBD with high probability (*P* < 0.0001). Clinically, in the pediatric setting of many patients with functional abdominal pain or irritable bowel syndrome [19–21] and in comparison to those relatively few IBD patients, elevated FL can help to define those patients who should undergo endoscopy and those who do not need invasive diagnostics.

## 5. Conclusion

Fecal lactoferrin is a reliable biomarker for active IBD in pediatric patients. At least for patients with CD, FL levels are correlated with disease severity as graded by the SES-CD. The serological marker CRP is less sensitive for active IBD. Thus, FL is a helpful biomarker to aid the early diagnosis of pediatric IBD, indicating the need for invasive diagnostics like endoscopy. It shows promise for monitoring the effectiveness of therapy because of its correlation of levels and degree of mucosal inflammation.

## Figures and Tables

**Table 1:** Patient and clinical characteristics.

Group	CD	UC	Control
*n*	21	15	20
Male/female	15/6	10/5	10/10
Mean/median age	13.2/13.4	10.9/12.7	11.9/12.6
Paris classification			
CD L1	4		
CD L2	4		
CD L3	13		
UC E3		1	
UC E4		14	
